# Supplementary material for: Transvaginal tru-cut biopsy versus ascitic fluid cytology in gynaecologic tumours: A comparative diagnostic study
Source: PLoS One. 2026 May 14;21(5):e0349602. doi: 10.1371/journal.pone.0349602 (PMC13175480; doi:10.1371/journal.pone.0349602)
Supplement: Attachment — Submitted filename: tru cut vs ascitic cytology.docx [file pone.0349602.s001.docx]

**Date:** Thursday, August 14th, 2025
**Journal:** PLOS ONE

**Dear Editor-in-Chief, Associate Editor, and Authors,**

Thank you for the opportunity to review the manuscript entitled *“Transvaginal tru-cut biopsy versus ascitic fluid cytology in gynecologic tumours: a comparative diagnostic study.”*

**Summary:**

In this retrospective study, the authors compared the adequacy, diagnostic accuracy, sensitivity, specificity, and safety of transvaginal ultrasound-guided tru-cut biopsy and ascitic fluid cytology in women with gynecologic tumours.

This study offers crucial comparative data, showing that ascitic fluid cytology is still a safe substitute in cases where biopsy is not possible, but tru-cut biopsy provides more diagnostic information. The results may affect gynecologic oncology diagnostic pathways, especially in settings with limited resources.

**Comments to Authors:**

**Abstract:**

- The abstract is concise and readable. The objectives, methods, and findings are well presented.
- The conclusion is appropriately stated based on the findings.

**Title:**

- The title is precise, clear, and informative. The title reflects the objectives and design of the study appropriately.

**Introduction:**

- The introduction provides a good background in terms of ascites and cytology in gynecologic malignancies.
- The rationale of comparing tru-cut biopsy and ascitic fluid cytology is well described.
- Nevertheless, a little more elaboration on the clinical significance of this comparison to patient care and decision-making, particularly in low-resource or outpatient practice, would be a welcome addition to the introduction.
- The introduction might also be stronger if it discussed the existing literature on this topic or stated clearly how the present study uniquely contributes to the knowledge base.

**Methods:**

- The retrospective design was clearly described, with proper comments regarding inclusion criteria, procedure, and data analysis.
- Statistical methodology was well described (sensitivity, specificity, PPV, NPV).
- Approval of ethics and waiver of informed consent was properly stated.
- The potential methodological limitation of collecting a small ascitic sample volume (20 mL) was noted. Most studies and international reporting systems have suggested collecting larger volumes, generally ≥50–75 mL for serous effusions, while some ascites-specific studies noted an ideal sample volume of 200 mL when classifying malignant ascites. Noted guidance suggests that taking multiple samples on days subsequently or using cell-block preparation can enhance diagnostic sensitivity with small volumes^1,2,3^. Addressing a rational for sampling only 20 mL (e.g. according to institutional protocol, keeping within patient tolerance for comfort, or resource limitations) would have strengthened the methods section and clarify how this may have influenced the results.

**Results:**

- The results are presented neatly, with tables to compare diagnostic values of both techniques.
- There is good reporting of the complication rate, which adds to the clinical applicability of the findings.
- However, subgroup analysis (e.g., by tumour type or stage) could be informative, as high-grade ovarian cancers predominated the cohort.

**Discussion:**

- The discussion is excellent in contrasting the findings with other literature and highlighting the benefits of tru-cut biopsy versus ascitic cytology.
- The authors have referred to the limitation of single-center study, small sample size, and retrospective design, which is welcome.
- Additional discussion regarding the clinician's practical rule of thumb of how to utilize tru-cut and how frequently just cytology would suffice would be helpful.

**Conclusion:**

- The conclusion answers the research question appropriately.
- The authors summarize the key findings clearly, but they may consider adding a forward-looking statement on how these findings could guide diagnostic protocols in gynecologic oncology.

**Overall Evaluation:** This is a well-conducted and clinically relevant study, which compares two important diagnostic examinations in gynecologic oncology. It is well written, well-reasoned, and adds valuable evidence to the literature. With minor revision to further bolster the clinical utility and defend some of the methodological choices, the manuscript is publishable.

**References:**
1) <https://doi.org/10.1002/cncy.22577>
2) Zhang F, Feng Z, Zhang Y, Liu Z, Sun X, Jin S. Determination of the optimal volume of ascitic fluid for the precise diagnosis of malignant ascites. Saudi J Gastroenterol. 2019 Sep-Oct;25(5):327-332. doi: 10.4103/sjg.SJG_547_18. PMID: 30900607; PMCID: PMC6784429.
3) https://doi.org/10.1159/000529855
